# Production and Evaluation of Lime Fertilizers with the Addition of Biomass Combustion Waste

**DOI:** 10.3390/ma18122732

**Published:** 2025-06-11

**Authors:** Sławomir Obidziński, Paweł Cwalina, Aneta Sienkiewicz, Małgorzata Kowczyk-Sadowy, Jolanta Piekut, Jacek Mazur, Michał Panasewicz

**Affiliations:** 1 Department of Agri-Food Engineering and Environmental Management, Bialystok University of Technology, Wiejska 45E, 15-351 Białystok, Poland; s.obidzinski@pb.edu.pl (S.O.); a.sienkiewicz@pb.edu.pl (A.S.); m.kowczyk@pb.edu.pl (M.K.-S.); j.piekut@pb.edu.pl (J.P.); 2 Department of Food Engineering and Machines, University of Life Sciences in Lublin, 28 Głęboka St., 20-612 Lublin, Poland; jacek.mazur@up.lublin.pl; 3 ROLPOL Ołdakowscy sp.j., 18-210 Szepietowo, Poland; michal.panasewicz@rolpol.com.pl

**Keywords:** fertilizers, innovation, sustainable agriculture, waste, biomass

## Abstract

The study identified the optimal material, e.g., raw composition and moisture content, and process parameters for the non-pressure agglomeration of carbonate lime combined with biomass waste, e.g., calcium sulfate (ECO-ZEC), post-production residue (PPR), and fly ash using a molasses-based binder. The chemical analysis revealed that the CaO content in the granules ranged from 34% to 52%, with the highest calcium concentration observed in formulations containing carbonate limestone. Among the waste-based additives, PPR exhibited a calcium content only 7% lower than that of pure carbonate lime, whereas ECO-ZEC and fly ash contained 20% and 30% less calcium, respectively. Due to the low MgO levels in the tested granules, they cannot be classified as calcium–magnesium fertilizers. Regarding heavy metal content, concentrations of cadmium and lead remained below the permissible regulatory limits. The highest levels of these elements were detected in the fly ash-enriched granules, consistent with the known chemical composition of this waste type. The tested waste materials ECO-ZEC, PPR, and fly ash demonstrated alkaline pH values ranging from 12.37 for fly ash and 12.28 for PPR to 8.84 for ECO-ZEC. The reference carbonate lime showed a slightly lower pH of 8.82. Mechanical strength testing indicated that the addition of PPR improved the mechanical resistance of the granules compared to the reference sample. Conversely, the inclusion of ECO-ZEC and fly ash reduced this parameter. Notably, granules containing fly ash and PPR exhibited prolonged disintegration times in water, suggesting their potential application as slow-release fertilizers. The findings of this study demonstrate that industrial waste materials generated from biomass combustion can serve as effective components in the production of innovative lime-based fertilizers. This innovative approach not only promotes the recycling of by-products but also supports the development of sustainable agriculture by reducing the environmental burdens associated with waste disposal and encouraging resource efficiency.

## 1. Introduction

The Council Directive 1999/31/EC of 26 April 1999 [1], commonly referred to as the Landfill Directive, introduces restrictions on waste disposal, including various types of ash. By imposing stringent operational and technical requirements, it indirectly encourages the development of alternative waste management strategies. In Poland, the main legislative act governing waste management is the Act of 14 December 2012 on Waste (*Journal of Laws* 2013, 21, as amended) [2], which outlines the principles for managing materials classified as waste.

A significant category of waste generated during electricity and heat production comprises energy waste, commonly referred to as the by-products of combustion (BPC). According to Stępień and Białecka [3], approximately 900–1000 million tons of BPC are produced annually worldwide, posing a considerable environmental challenge. The European Union contributes roughly 100 million tons, while the United States generates around 130 million tons annually [3]. In Poland, annual production includes 9 million tons of ash–slag mixtures, 4 million tons of fly ash, and 3.8 million tons of mixtures combining fly ash and solid residues from calcium-based flue gas desulfurization. In 2018 alone, 17 million tons of BPC were generated, ranking third in total waste production in Poland [4]. Approximately 60% of this waste is recovered, while the remainder is landfilled, highlighting the urgency of identifying new management pathways [3].

With the EU’s push toward increasing the share of renewable energy sources, biomass combustion is expected to rise, further intensifying the generation of biomass ash that requires management. Currently, global biomass combustion and co-combustion processes produce an estimated 480 million tons of ash, associated with an annual biomass usage of 7 billion tons [5]. While coal ash is widely utilized in the construction industry, particularly in cement manufacturing [6,7], the application of biomass ash is more limited due to its distinct chemical composition. Of particular concern is its phosphorus content, which often exceeds limits set by EN 450-1 [8], resulting in delayed hydration, extended setting times, and reduced concrete strength [9].

In recent years, growing interest has been in utilizing biomass combustion ash in agriculture as a fertilizer [10,11]. Key advantages include its strong soil alkalizing potential—particularly for acidic soils; its high content of essential minerals such as manganese, zinc, and copper; and its relatively low risk of heavy metal contamination in soils and crops [12,13,14]. Moreover, its chemical composition includes macronutrients like potassium, calcium, and magnesium [13], supporting its use in soil reclamation and fertilization applications [15,16]. Biomass ash also generally contains low levels of polycyclic aromatic hydrocarbons (PAHs), often below EU regulatory limits [17,18], and may be suitable for use in organic farming or forest soil enrichment due to its low heavy metal content [14]. Overall, returning biomass ash to agricultural soils is considered both an ecological and sustainable management strategy. Additionally, such ash is inexpensive and often freely available to farms operating biomass combustion systems.

Fly ash, another combustion by-product, is also produced in large quantities. In 2018, Poland generated 2.4 million tons of fly ash [4], with over 100,000 tons sent to landfills due to oversupply. Currently, 25.5 million tons of fly ash remain in storage. This material exhibits pozzolanic properties and is used in road construction, mining, and various engineering applications [19,20]. It also finds application as a filler in polymer processing (e.g., polypropylene, PVC, PE, and PET) [21]. Fly ash composition varies significantly depending on the fuel source: coal ash is rich in SiO_2_ and Al_2_O_3_, whereas lignite ash contains more calcium and magnesium [22]. The higher content of these nutrients makes lignite ash an effective liming agent and a valuable source of macro- and micronutrients. However, its use is hindered by particle aggregation and the absence of formal regulatory approval. Currently, it can be added to compost or used for sewage sludge sanitation and conditioning [23].

Compared to bottom ash, fly ash generally offers greater nutrient availability for plants, making it more suitable for agricultural and soil improvement applications [11,14,24]. Its high water absorption capacity improves soil porosity and water retention, enhancing root penetration [25,26]. Moreover, fly ash increases soil pH and positively affects the properties of organic matter [27,28]. Beyond physical and chemical improvements, fly ash may also benefit crop development [29,30,31]. However, due to variable chemical compositions, careful monitoring is essential, particularly regarding soil pH, salinity, and the potential accumulation of trace elements [32].

Studies have explored combining biomass ash with poultry manure [33], carbonated lime with cattle slurry [34], and sugar industry by-products [35]. Biomass fly ash (BFA) has also been used with distilled water, biological sludge, and composted sludge [36]. Nevertheless, limited research has addressed the integration of biomass waste with granulated lime fertilizers. Some work has examined the co-granulation of bio-ash with lime kiln dust [37,38] or in combination with sewage sludge [39]. Other innovative formulations include non-pressure agglomeration with sulfur gypsum and limestone flour wetted with lignosulfonates [40] or crushed stone waste used in sorghum fertilization [41]. Paleckienė et al. [42] produced calcium-enriched fertilizers using sugar lime granules. Ławińska et al. [43] granulated leather industry waste (tanning shavings) using sodium silicate, dolomite, and gypsum. Obraniak et al. [44] assessed the impact of bentonite, water, and sodium silicate on fly ash granule durability via disc granulation. Patents have also been granted for the production of granulated lime fertilizers using both natural raw materials and industrial by-products [45,46,47].

Soil liming is a commonly used practice, especially on acidic soils, which are typical of many agricultural regions—particularly in Poland and other countries with a similar climate. Regular liming is essential for maintaining optimal soil pH, which supports better plant growth and more efficient use of mineral fertilizers [48]. This study aligns with ongoing efforts to develop innovative granulated liming fertilizers enriched with nutrients, aiming for products with desirable agronomic and physical characteristics—namely, non-clumping, free-flowing granules that are easy to store and apply mechanically. The main objective was to identify optimal material (raw composition, moisture content) and process parameters for the non-pressure agglomeration of carbonate lime combined with biomass waste and a molasses-based binder. The study evaluates the impact of three types of industrial biomass waste and a molasses binder on the final granules’ ash content, macro- and microelement composition (including heavy metals), and water absorption capacity. The findings demonstrate the feasibility of producing a high-quality granular fertilizer, supporting sustainable waste management, and enhancing fertilization practices in agriculture.

## 2. Materials and Methods

### 2.1. Materials

**Figure 1:**
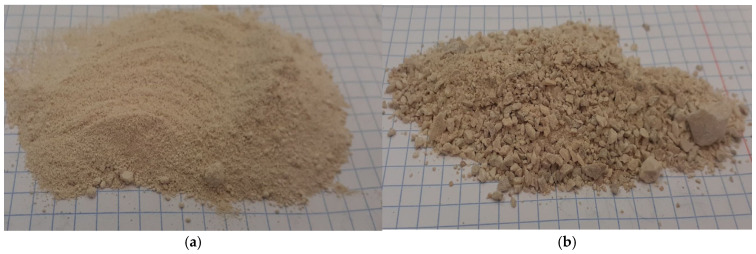
The limestone used during the study: (**a**) ground and (**b**) unground.

The primary raw material used in this study was carbonate limestone (Figure 1), provided by ROLPOL Ołdakowscy Spółka Jawna, located in Szepietowo, Podlaskie Voivodeship, Poland.

During the non-pressure agglomeration process, waste materials were incorporated into the limestone. These materials, also supplied by ROLPOL, included the following:

- ECO-ZEC (calcium sulfate)—an industrial by-product containing calcium sulfate dihydrate, obtained from a flue gas desulfurization system;
- PPR (post-production residue)—a post-reaction product derived from the semi-dry flue gas desulfurization process in boilers, produced by ENEA Ciepło, Białystok Combined Heat and Power Plant;
- Fly ash—a combustion by-product from biomass, sourced from the same ENEA Ciepło facility.

**Figure 2:**
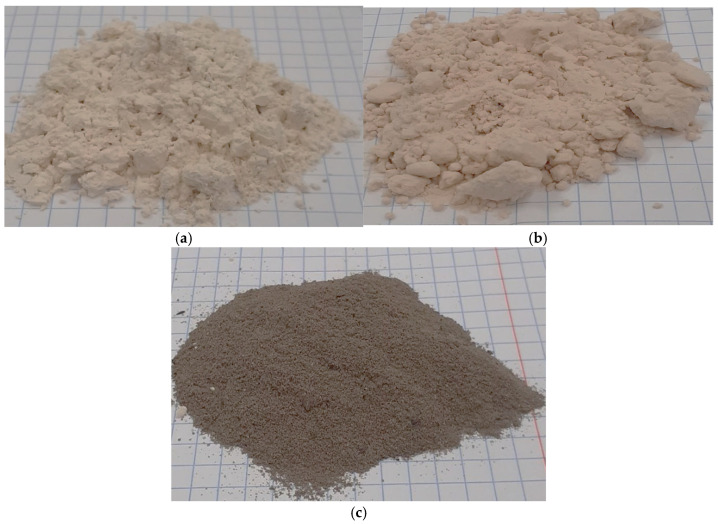
The waste materials used during the study: (**a**) ECO-ZEC, (**b**) PPR, and (**c**) fly ash.

The waste materials used in the study are presented in Figure 2. Additionally, molasses—also supplied by ROLPOL—was used as a binder in the agglomeration process.

### 2.2. Moisture Content Determination

The moisture content of the raw materials limestone, ECO-ZEC, PPR, and fly ash, as well as of the agglomerated products, was determined according to the PN-EN ISO 18134-3:2023-12 standard [49], using an AXIS ASG laboratory moisture analyzer (AXIS, Gdańsk, Poland). For each material, five 5 g samples were analyzed. The samples were dried at 105 °C, and the average of the obtained values was reported as the final moisture content.

### 2.3. Particle Size Distribution Analysis

The particle size distribution of the raw materials limestone, ECO-ZEC, PPR, and fly ash was determined through sieve analysis using an LPz-2e programmable sieve shaker (Multiserv Morek, Marcyporęba, Poland) and a standard sieve set. Sieves with mesh sizes of 4.0 mm, 2.0 mm, 1.0 mm, 0.5 mm, 0.25 mm, 0.125 mm, and 0.063 mm were used. The analysis was performed by PN-R-64798:2009 [50].

### 2.4. Bulk Density Determination

Bulk density was determined for all raw materials—limestone, ECO-ZEC, PPR, and fly ash—using a 407.5 cm^3^ metal cylinder, a laboratory scale (AX324M, OHAUS Europe GmbH, Nänikon, Switzerland), and a steel scraper. The procedure followed the method described in reference [51].

### 2.5. Fertilizer Production via Non-Pressure Agglomeration

**Figure 3:**
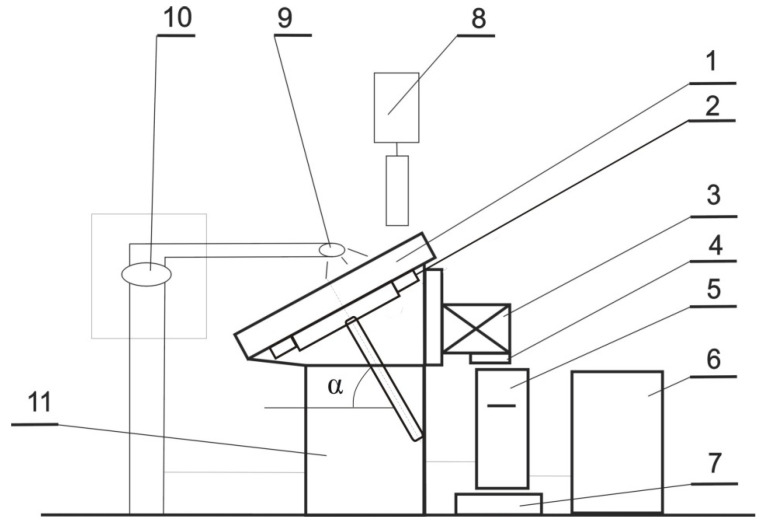
Schematic diagram of the test stand for plate granulation: (1) replaceable granulation plate, (2) gear mechanism, (3) electric motor with belt transmission, (4) frequency converter, (5) moisturizing liquid tank, (6) compressor, (7) scale, (8) raw material dispenser, (9) spray nozzle, (10) rotameter, (11) supporting structure, and (α) plate inclination angle.

The production of fertilizer granules from carbonate limestone combined with selected waste materials ECO-ZEC, PPR, and fly ash was carried out using the non-pressure agglomeration method at the experimental research station. The schematic of the production setup is shown in Figure 3.

The non-pressure agglomeration process was conducted under controlled conditions with the following constant operational parameters:

- Granulation plate inclination angle: 65°;
- Blade inclination angle within the plate granulator: 45°.

Based on preliminary trials, the following operating parameters of the granulation system were selected:

- Granulation plate rotational speed: 8 rpm;
- Material residence time in the granulator: 9 min;
- Granulation liquid droplet size: mist (fine spray).

These settings were maintained throughout the study in order to investigate the influence of different mixture compositions on the granulated fraction yield and the quality of the final product.

The granulation tests were carried out using specific compositions of ground carbonate lime and selected waste materials provided by ROLPOL. Detailed formulations used in the experimental setup are presented in Table 1.

Following the granulation process, the obtained granules were transferred onto metal trays and placed on the shelves of a convection dryer (BMT Medical Technology s.r.o., Brno, Czech Republic). The drying process was carried out for 24 h at a controlled temperature of 80 °C and monitored using a contact thermometer (Testo SE & Co. KGaA, Titisee-Neustadt, Germany).

### 2.6. Determination of Macroelements

The contents of sodium (Na), potassium (K), magnesium (Mg), and calcium (Ca) were determined using atomic absorption spectrophotometry (AAS). This technique enabled the direct quantification of macroelements in the solutions obtained from previously mineralized samples. The analyses were performed using a Thermo Scientific iCE 3300 flame atomic absorption spectrometer (Thermo Scientific, Waltham, MA, USA). Calibration curves were established based on standard solutions of Na, K, Mg, and Ca in concentrations ranging from 0.5 to 100 mg·L^−^^1^. Subsequently, the macroelement concentrations in the tested mineralizates were determined.

### 2.7. Determination of Microelements and Heavy Metals

The concentrations of selected elements, including aluminum (Al), phosphorus (P), chromium (Cr), manganese (Mn), iron (Fe), cobalt (Co), nickel (Ni), copper (Cu), zinc (Zn), arsenic (As), cadmium (Cd), lead (Pb), and mercury (Hg), were measured using inductively coupled plasma mass spectrometry (ICP-MS). The analyses were conducted with an Agilent Technologies 8800 Triple Quadrupole ICP-MS system (Agilent Technologies, Santa Clara, CA, USA). ICP-MS is recognized as a state-of-the-art analytical technique for trace element detection, offering a wide linear dynamic range, low detection limits, high throughput, and the capability for simultaneous multi-element analysis. The method involves ionization of the sample in plasma (at approximately 6000–10,000 K), followed by ion separation via a mass analyzer and detection based on ion stream intensity. The detection limits achieved are influenced by the target element, matrix composition, and sample preparation technique.

### 2.8. Determination of Granule Crushing Strength

**Figure 4:**
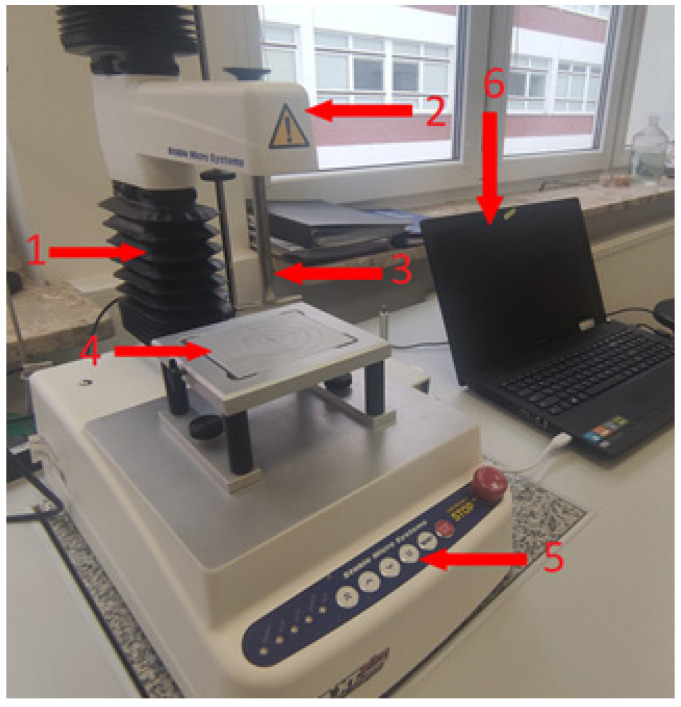
Station for determining crushing strength of granules [own study]: (1) overhead crane, (2) load cell, (3) measuring tip, (4) measuring platform, (5) controller with switch, and (6) computer with Exponent 6.1.27.0 software.

The mechanical strength of the produced granules was evaluated by measuring their crushing resistance. A dedicated test stand for assessing the crushing strength of granular materials was employed for this purpose (Figure 4).

During the crushing strength test of the granulate, the following Exponent software settings options were used:

- Mode: Return to Start;
- Pre-Test Speed: 10 mm·s^−1^;
- Test Speed: 0.5 mm·s^−1^;
- Post-Test Speed: 10.0 mm·s^−1^;
- Distance: 2 mm;
- Tools: Measuring tip for granulate testing, Heavy Duty Platform (HDP/90).

### 2.9. Determination of the Disintegration Time of Fertilizer Granules in Water

The disintegration time of the fertilizer granules in water was assessed to evaluate their solubility and potential behavior as slow- or fast-release fertilizers. The methodology used for this determination was developed internally by ROLPOL and has been applied consistently in their production and quality control processes.

**Figure 5:**
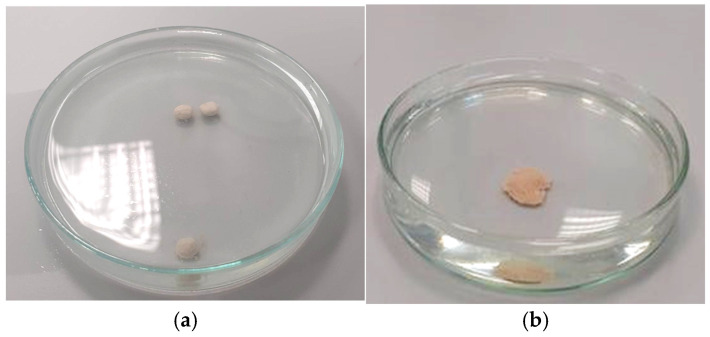
Station for determining solubility of granules: (**a**) with the granule just after it has been thrown into water, and (**b**) with the granule after it has disintegrated in water.

The experimental setup used for the test is presented in Figure 5. The granules were immersed in distilled water at room temperature, and the time required for complete physical disintegration of the granules was recorded.

This parameter is crucial for assessing the release rate of nutrients from the fertilizer and provides insight into the granules’ suitability for different soil conditions and crop requirements. A prolonged disintegration time may indicate the potential of the product to act as a slow-release fertilizer, contributing to improved nutrient management and reduced environmental impact.

### 2.10. Determination of pH Value

The pH values of the raw materials and produced granulates were determined using the potentiometric method. Measurements were conducted in aqueous suspensions prepared by mixing the sample with distilled water at a 1:10 solid-to-liquid ratio. The pH was recorded using a calibrated pH meter under laboratory conditions. This method is widely recognized for its accuracy and reproducibility in determining the acid-base properties of solid materials, including those intended for agricultural use.

### 2.11. Statistical Analysis

All results are expressed as mean values ± standard deviation (SD), calculated from three independent replicates. To identify underlying patterns and groupings within the dataset, hierarchical cluster analysis (HCA) was employed. The analysis was based on Euclidean distance as the dissimilarity measure and Ward’s linkage method as the agglomeration strategy.

The resulting dendrogram effectively classified the samples into five distinct clusters, reflecting the similarities in their physicochemical and mechanical properties. Statistical analyses were performed using Statistica 13.3 software (TIBCO Software Inc., Palo Alto, CA, USA).

HCA was selected due to its robustness in handling multivariate datasets, making it particularly suitable for complex material characterization. It enables objective classification without the need to predefine the number of clusters, thereby offering valuable insight into the relationships between the chemical composition and performance characteristics of the tested granulated fertilizers.

## 3. Results and Discussion

### 3.1. Moisture Content of Tested Materials

Table 2 presents the measured moisture content of the input materials, including limestone and selected industrial by-products—ECO-ZEC, PPR, and fly ash. These values provide essential insights into the hygroscopic properties of the raw materials before pelletization.

Based on the conducted measurements, the limestone utilized in the non-pressure agglomeration process exhibited relatively low moisture levels, with values of 0.39% for ground lime and 0.36% for unground lime. In contrast, the examined waste materials demonstrated higher moisture content: 0.40% for fly ash, 1.82% for PPR, and 8.17% for ECO-ZEC. According to the authors’ practical experience and insights from the literature, optimal conditions for effective pellet formation are typically achieved when the moisture content of limestone ranges between 5% and 15%.

### 3.2. Granulometric Distribution of Tested Materials

Table 3 shows the results of the granulometric distribution tests (the percentage of the material retained on each sieve fraction) for the raw materials: limestone and waste materials ECO-ZEC, PPR, and fly ash.

The sieve analysis revealed that ground limestone exhibited the highest particle share in the 0.25 mm (58.31%) and 0.125 mm (27.03%) fractions. In contrast, unground limestone was primarily concentrated in the coarser fractions—1.00 mm (42.05%) and 0.50 mm (19.39%).

For the PPR, the dominant fractions were 0.50 mm (63.06%) and 0.25 mm (29.14%). Notably, partial agglomeration of particles occurred during the analysis, particularly on the 2.00 mm sieve, which influenced the distribution across subsequent sieves.

The granulometric profile of ECO-ZEC showed that the entire sample was retained within three sieve fractions: 0.50 mm (52.79%), 1.00 mm (26.50%), and 2.00 mm (20.70%). Similar to PPR, this distribution pattern is attributed to the partial agglomeration of particles on the sieves.

Fly ash demonstrated a significantly finer particle size distribution, with the majority of material found in the 0.25 mm (48.14%), 0.125 mm (31.32%), and 0.063 mm (19.96%) fractions.

**Figure 6:**
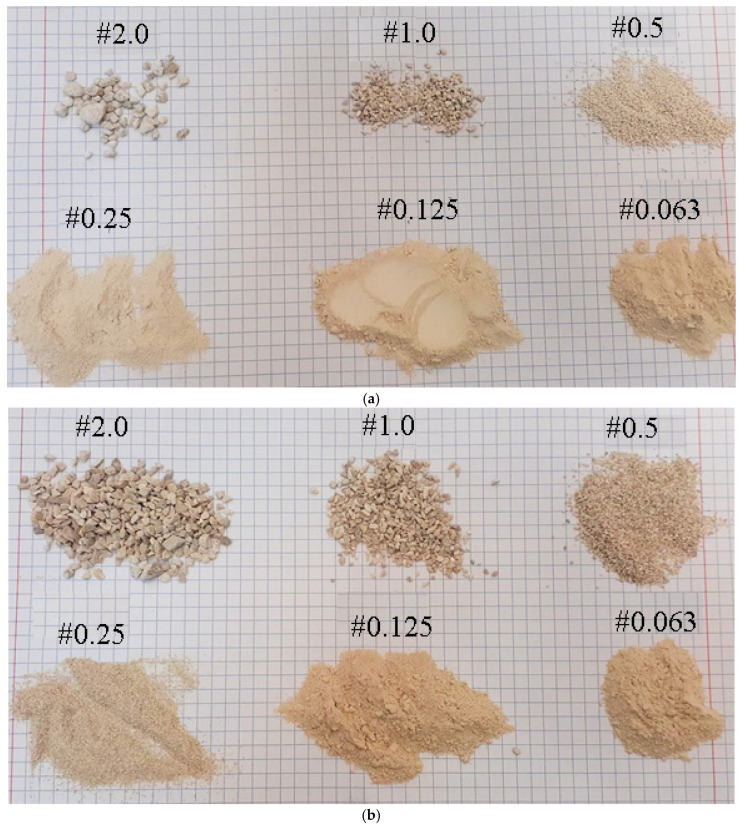
Individual fractions of raw materials subjected to sieve analysis: (**a**) ground lime and (**b**) unground lime.

**Figure 7:**
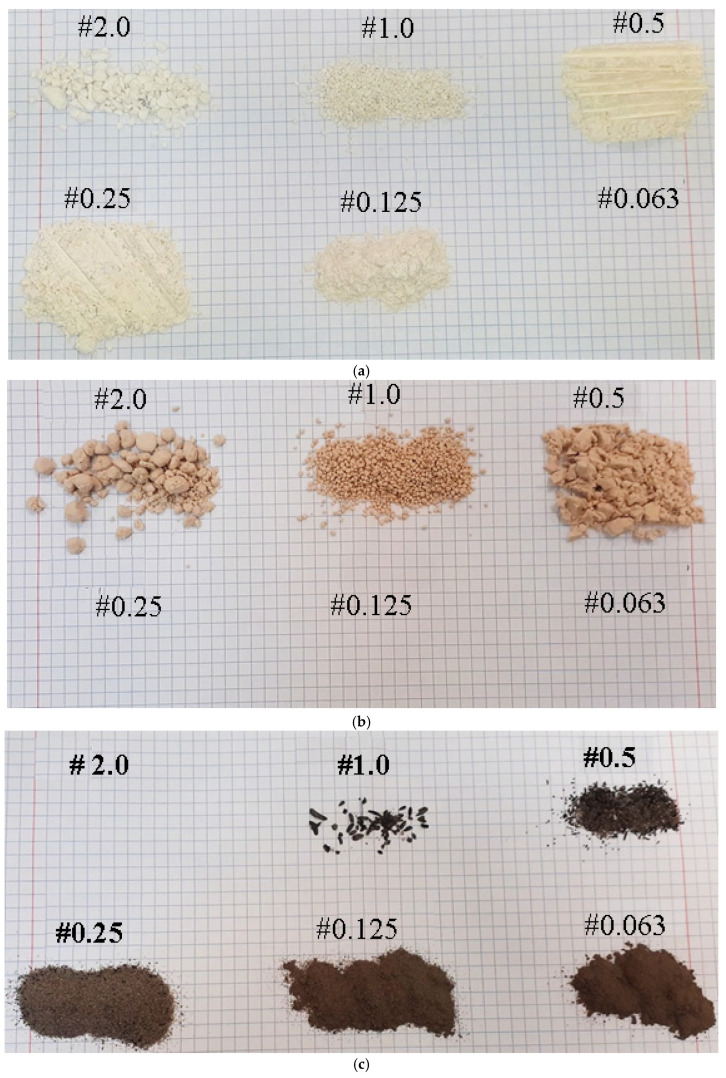
Individual fractions of waste additives subjected to sieve analysis: (**a**) PPR, (**b**) ECO-ZEC, and (**c**) fly ash.

Figure 6 and Figure 7 provide a graphical representation of the particle size distributions for both the raw limestone and the examined waste materials, offering a comparative overview of their granulometric characteristics.

### 3.3. Macroelement Content

Table 4 summarizes the concentrations of selected macroelements—namely, sodium (Na), magnesium (Mg), calcium (Ca), and sulfur (S)—along with their corresponding oxide forms (Na_2_O, MgO, CaO, and SO_3_), determined in both the primary raw material limestone and the industrial by-products ECO-ZEC, PPR, and fly ash. This compositional analysis provides key insights into the chemical characteristics relevant to their potential application in agglomeration processes.

Based on the conducted analyses, the highest sodium (Na) content was identified in fly ash, while the lowest concentration was recorded in ECO-ZEC. In terms of magnesium (Mg), limestone exhibited the greatest amount, whereas fly ash contained approximately half as much. ECO-ZEC showed the lowest magnesium content among the tested materials.

A similar distribution trend was observed for calcium (Ca). The calcium content in limestone reached 38.44%, making it the most calcium-rich material. In comparison, PPR exhibited about 7% less calcium, ECO-ZEC approximately 20% less, and fly ash over 35% less than limestone.

Sulfur (S) content was minimal in limestone (0.0002%) and remained relatively low in fly ash (0.03%). In contrast, PPR presented a significantly elevated sulfur concentration of 15.26%, which corresponds to 38.14% when expressed as sulfur trioxide (SO_3_). The highest sulfur content was recorded in ECO-ZEC, amounting to 20.82%, equivalent to 52.04% SO_3_.

The chemical composition of fly ash derived from biomass combustion varies considerably depending on both the biomass type and combustion conditions [11,52,53]. Ash produced from coal combustion is typically rich in silicon dioxide (SiO_2_, ~50%) and aluminum oxide (Al_2_O_3_, >20%). In contrast, fly ash from lignite combustion contains higher concentrations of calcium (9.21%), magnesium (0.92%), and sulfur (0.67%) compared to ash from hard coal, which contains only 1.42% Ca, 0.45% Mg, and 0.31% S [54].

Jarosz-Krzemińska and Poluszyńska [55] investigated fly ash originating from wood combustion in fluidized bed boilers. Their findings revealed a substantial presence of calcium oxide (CaO), ranging from approximately 12.9% to 26.5%, due to the inherently high calcium content in wood tissues, particularly bark. Magnesium oxide (MgO) was detected at levels of 3–4%, while phosphorus pentoxide (P_2_O_5_) ranged from 2% to 4.6%. In the case of non-wood plant biomass (e.g., straw, corn, sunflower husks, or palm kernels), significantly higher concentrations of potassium, phosphorus, and magnesium were observed. For example, straw ash may contain over 66% SiO_2_, sunflower husk ash up to 31% K_2_O, and corn ash as much as 42% P_2_O_5_ and over 10% MgO [11].

Granules produced from mixtures of limestone and the waste materials ECO-ZEC, PPR, and fly ash are characterized by a CaO content ranging from 34% to 52% (Table 5). Therefore, according to the “Regulation of the Minister of Economy of 8 September 2010” [56], on the method of packaging mineral fertilizers, the placement of information on fertilizer components on these packages, the method of testing mineral fertilizers, and the types of agricultural lime, they can be classified as agricultural lime of type 07—derived from by-products. Those with higher calcium oxide content can be classified as agricultural lime derived from the processing of limestone rocks, types 05 and 06 (Table 5).

The magnesium oxide (MgO) content in the tested granules ranges from 0.6% to 1.8%. Due to the low MgO content, the produced granules cannot be classified as agricultural lime containing magnesium. The granules also showed a low sodium content, below 0.1%. When comparing the granules in terms of sodium content, it was found to be highest in the mixtures containing fly ash and lowest in those with ECO-ZEC. The granule obtained from a mixture of limestone with 70% ECO-ZEC waste lime, due to its sulfur trioxide (SO_3_) content exceeding 35%, can additionally be considered a fertilizer containing secondary nutrients.

### 3.4. Microelement Content and Heavy Metals

Table 6 presents the results of the analysis of chromium (Cr), manganese (Mn), iron (Fe), cobalt (Co), nickel (Ni), copper (Cu), zinc (Zn), arsenic (As), cadmium (Cd), and lead (Pb) content in granules produced from limestone and the waste materials ECO-ZEC, PPR, and fly ash supplied by the company ROLPOL. According to the “Regulation of the Minister of Agriculture and Rural Development of 18 June 2008” [57], on the implementation of certain provisions of the Fertilizers and Fertilization Act, the permissible level of contaminants in mineral fertilizers must not exceed 8 mg·kg^−^^1^ CaO for cadmium and 200 mg·kg^−^^1^ CaO for lead. In the tested granules, cadmium content ranged from 0.40 to 2.32 mg·kg^−^^1^ CaO, and lead content from 3.48 to 41.41 mg·kg^−^^1^ CaO, meaning that the permissible levels of heavy metals were not exceeded. The highest concentrations of these elements were found in granules with added fly ash, which is due to the fact that fly ash contained the highest amounts of cadmium and lead among the analyzed raw materials. The granulate produced from limestone with the addition of 35% fly ash and 65% limestone had the highest content of chromium (0.0016%), manganese (0.0652%), iron (0.332%), cobalt (0.0000992%), zinc (0.0140%), cadmium (0.000868%), and lead (0.00147%). The granulate produced with the addition of 70% ECO-ZEC had the lowest content of chromium (0.000507%), manganese (0.00431%), iron (0.0609%), cobalt (0.00000337%), zinc (0.00234%), and arsenic (0.00000176%).

The microelement with the highest concentration, both in raw and waste materials, was iron, with contents ranging from 0.061% to 0.332%. Based on the research by Smołka-Danielowska and Jabłońska [58], fly ash from woody biomass contains from 100 to 300 mg·kg^−^^1^ of zinc, from 30 to 80 mg·kg^−^^1^ of copper, and from 5 to 20 mg·kg^−^^1^ of lead. On the other hand, the content of cadmium ranges from 0.5 to 2.0 mg·kg^−^^1^, nickel from 10 to 25 mg·kg^−^^1^, chromium 5–15 mg·kg^−^^1^, arsenic 1–3 mg·kg^−^^1^, and mercury 0.1–0.5 mg·kg^−^^1^. Despite the presence of heavy metals, their concentrations usually do not exceed the permissible standards for the use of ash as organo-mineral fertilizers. However, it is necessary to individually test each ash sample for its chemical composition and possible toxicity. In a study conducted by Jukić et al. [59], it was found that the content of heavy metals in fly ash from woody biomass is generally low; however, in some samples, the levels exceeded the permissible limits for fertilizers or soil improvers. For example, the lead (Pb) content in woody biomass ash may range from 20 to 40 mg·kg^−^^1^, while the cadmium (Cd) content ranges from 0.5 to 1.5 mg·kg^−^^1^.

### 3.5. Bulk Density of Tested Materials

**Figure 8:**
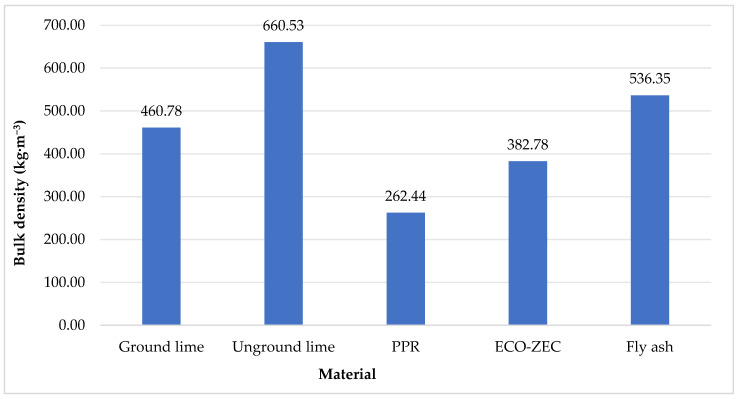
Bulk density of the raw materials and waste additives used during the study.

Figure 8 shows the results of the bulk density tests for the raw materials: limestone and the waste materials ECO-ZEC, PPR, and fly ash.

Based on the tests, it was observed that among the tested limestones, unground lime exhibited a significantly higher bulk density value of 660.53 kg·m^−^^3^ compared to ground lime, which had a bulk density of 460.78 kg·m^−^^3^. Among the waste additives, fly ash demonstrated the highest bulk density value of 536.35 kg·m^−^^3^. In contrast, ECO-ZEC lime had a lower bulk density value of 382.78 kg·m^−^^3^, while PPR exhibited the lowest bulk density value of 262.44 kg·m^−^^3^.

### 3.6. Fertilizing Properties of the Produced Granulates

**Figure 9:**
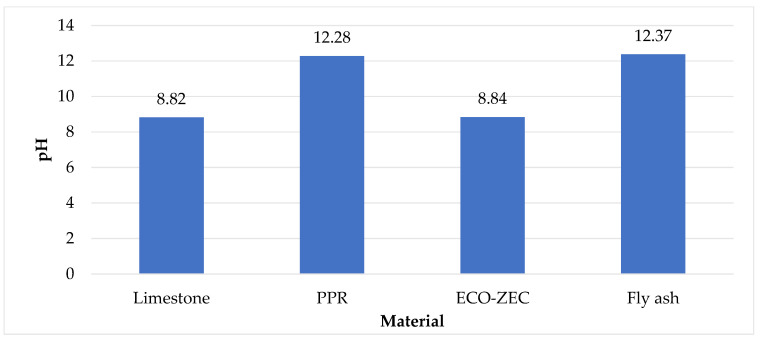
pH values of raw materials, including carbonate limestone and waste materials ECO-ZEC, PPR, and fly ash supplied by ROLPOL.

**Figure 10:**
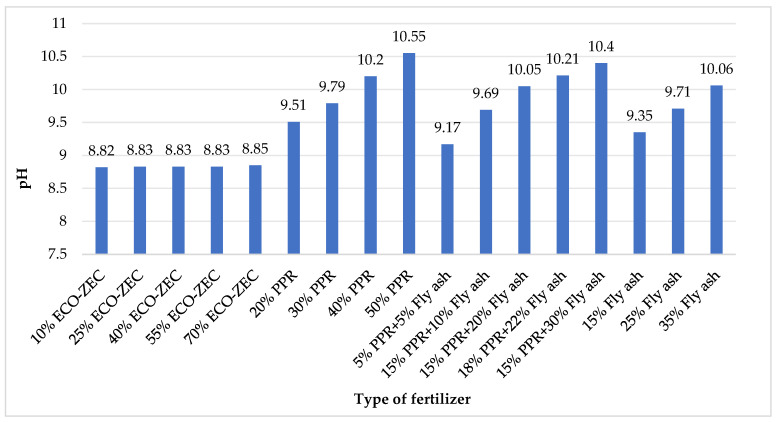
pH values of granulates obtained from mixtures of calcium carbonate and waste materials ECO-ZEC, PPR, and fly ash supplied by ROLPOL.

Figure 9 presents the pH values of the raw materials, including limestone and waste materials ECO-ZEC, PPR, and fly ash value, while Figure 10 shows the pH values of the granulates produced from mixtures of carbonate lime and waste materials ECO-ZEC, PPR, and fly ash supplied by ROLPOL and used in the tests.

The analyzed waste materials supplied by ROLPOL—ECO-ZEC, PPR, and fly ash—exhibited alkaline pH values. Fly ash showed the highest alkalinity with a pH of 12.37, followed closely by PPR at 12.28. These values are comparable to those reported by Stankowski et al. [60], who found that biomass ash exhibited a pH of 13.00. Similarly, Balcarik et al. [61] reported that waste from biomass combustion had a pH of 11.2.

The ECO-ZEC material demonstrated a moderately alkaline reaction, with a pH of 8.84, whereas carbonate lime exhibited a slightly lower pH of 8.82. These results are slightly higher than those reported by Feng et al. [62], where CaCO_3_-based calcium fertilizer presented a pH of 7.77.

The high pH values observed in the raw materials confirm the alkaline character of the resulting fertilizer granulates. When applied to soil, these materials are not expected to decrease soil pH. On the contrary, their application aims to neutralize soil acidity, reduce the harmful effects of aluminum and hydrogen ions, enhance nutrient availability, stimulate soil microbial activity, increase humus content, and improve overall soil structure and fertility [63]. It is important to note, however, that the optimal availability of most macronutrients occurs within a soil pH range of 6.5 to 8.5 [64,65]. Therefore, using the produced granulates offers a potential means for maintaining or adjusting soil pH to within this favorable range.

**Figure 11:**
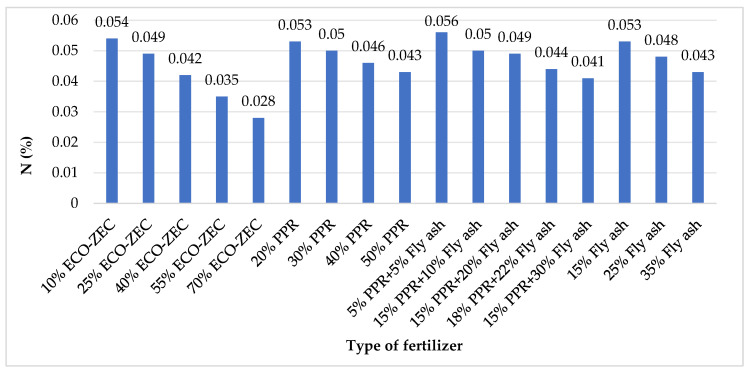
Nitrogen content in granules obtained from mixtures of carbonate lime and waste materials ECO-ZEC, PPR, and fly ash supplied by ROLPOL.

Figure 11 presents the nitrogen (N) content in the analyzed granulates, indicating their fertilizing potential.

The nitrogen content in granules formulated with carbonate lime and ECO-ZEC ranged from approximately 0.03% to 0.05%, while in those with the addition of PPR, the nitrogen content varied between 0.04% and 0.05%. The highest nitrogen levels were observed in the granules containing fly ash, where values ranged from 0.04% to approximately 0.06% (Figure 11). Despite these differences, the overall nitrogen content in the tested granulates remains low compared to selected organic fertilizers, such as manure (0.45%), compost (0.5%), and slurry (0.3%) [66]. For comparison, ashes derived from sewage sludge incineration typically exhibit significantly higher nitrogen contents, ranging from 1.5% to 2.3% [67,68].

**Figure 12:**
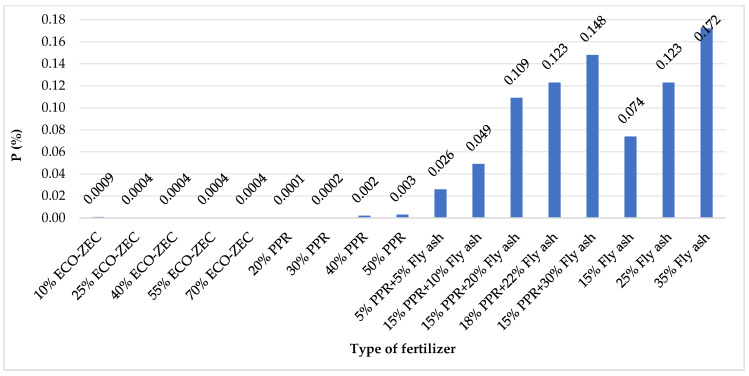
Phosphorus content in granulates obtained from mixtures of carbonate limestone and waste materials ECO-ZEC, PPR, and fly ash supplied by ROLPOL.

Among the analyzed granulates, the highest phosphorus (P) content was recorded in mixtures containing fly ash (Figure 12). This finding corresponds with the chemical composition of the raw materials, as fly ash exhibited the highest phosphorus and potassium levels among the tested inputs. When expressed as phosphorus pentoxide (P_2_O_5_), the phosphorus content in the granulates with ash ranged from 0.10% to 0.34%. According to Wierzbowska et al. [69], soil fertilization with ash derived from the combustion of energy willow and Virginia fanwort resulted in phosphorus contents of 3.3% and 1.66%, respectively, indicating that such ashes can be a rich source of this macronutrient.

**Figure 13:**
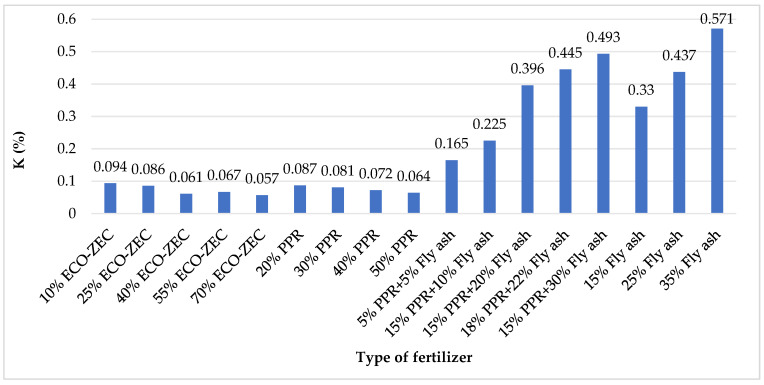
Potassium content in granulates obtained from mixtures of carbonate limestone and waste materials ECO-ZEC, PPR, and fly ash supplied by ROLPOL.

The highest potassium (K) content was recorded in mixtures also containing fly ash (Figure 13). When assessing the potassium content in terms of potassium oxide (K_2_O), it can be concluded that the fertilizer granulates containing fly ash are characterized by relatively low levels, ranging from 0.2% to 0.6% K_2_O. These values are comparable to those found in certain organic fertilizers, such as liquid manure (0.3% K_2_O) and compost (0.1–1.0% K_2_O). According to Stankowski et al. [60], ash derived from biomass combustion typically contains an average potassium content of 5.55%, whereas ash from sewage sludge combustion contains significantly lower levels—approximately 1.55% K_2_O [70].

### 3.7. The Results of the Research on the Non-Pressure Agglomeration Process

Table 7 presents the results of non-pressure agglomeration tests conducted on mixtures of carbonate lime and waste materials ECO-ZEC, PPR, and fly ash supplied by ROLPOL. The table also includes the compressive strength [N], bulk density [kg·m^−^^3^], and granulometric distribution [%] of the obtained granulates.

#### 3.7.1. Granulate Compressive Strength

Based on the test results (Table 7), very high compressive strength values were observed for granulates containing PPR as an additive to ground carbonate limestone. An increase in the PPR content from 30% to 50% in the mixture resulted in an increase in granule crushing strength by approximately 51%, from 9.25 N to 14.00 N. The compressive strength of granulates containing 30% PPR (9.25 N) was comparable to that of the reference granulate (10.60 N). With 40% PPR, the strength slightly exceeded the reference (10.76 N), while with 50% PPR, it was significantly higher (14.00 N).

In contrast, much lower compressive strength values were recorded for granulates with ECO-ZEC lime added to ground carbonate limestone. Increasing the ECO-ZEC content from 10% to 70% resulted in a decrease in compressive strength by approximately 9%, from 5.11 N to 4.65 N. The crushing strength of granulates with ECO-ZEC (regardless of dosage level) remained significantly lower than that of the reference granulate (10.60 N).

Much higher fertilizer compressive strengths were reported by Miastkowski et al. [71], who granulated a fertilizer mixture using bentonite as a binder. Depending on the bentonite concentration, the resulting granules showed compressive strength values ranging from 5.25 N to 46.15 N. Similarly, Malinowski et al. [72] studied fertilizers made from waste derived from magnesium nitrate production, which exhibited compressive strength ranging from 10.0 N to 70.2 N.

#### 3.7.2. Bulk Density of Granulate

Based on the experimental results (Table 7), relatively high bulk density values were observed for granulates obtained from ground carbonate lime supplemented with either PPR or ECO-ZEC lime. Increasing the PPR content from 30% to 50% in the mixture led to a modest rise in bulk density, approximately 3%, from 1054.74 to 1084 kg·m^−^^3^. Conversely, the addition of ECO-ZEC lime at increasing proportions (from 10% to 70%) resulted in a gradual decrease in bulk density by around 6%, from 1108.84 to 1047.07 kg·m^−^^3^.

The highest bulk density values were recorded in formulations combining fly ash and PPR. A mixture containing 5% fly ash and 5% PPR yielded a bulk density of 1129.77 kg·m^−^^3^, which was approximately 7% lower than the reference granulate (1210.78 kg·m^−^^3^). Increasing the PPR content to 15% in a mixture with 20% fly ash slightly improved the bulk density to 1172.43 kg·m^−^^3^. Further increasing the fly ash content to 30%, while maintaining a constant 15% PPR addition, resulted in a bulk density of 1159.72 kg·m^−^^3^.

In contrast, the addition of fly ash alone (25%) to ground lime produced granulates with slightly lower bulk density than the reference material. Overall, granulates containing PPR, ECO-ZEC lime, or fly ash showed reduced bulk density compared to the reference granulate, likely due to lower granule size uniformity in the modified formulations.

Bulk density of fertilizers varies widely depending on the raw materials used. Mudryk et al. [73] reported bulk densities ranging from 648.3 to 794.5 kg·m^−^^3^ for granulates based on chalk. Balla et al. [74] observed higher values for mineral fertilizers, ranging from 924.13 to 1070.72 kg·m^−^^3^. Comparable results were also obtained by Mudryk et al. [75] in the granulation of biomass ash blended with post-fermentation fertilizers, where bulk densities ranged from 669.13 to 890.36 kg·m^−^^3^.

#### 3.7.3. Granulometric Distribution of Fertilizer Granules

Granulometric analysis (Table 7) revealed that the dominant particle size fraction in fertilizer granulates composed of ground lime and waste additives ECO-ZEC, PPR, and fly ash was 8 mm. The 4 mm fraction constituted the second largest share. Smaller fractions—2 mm, 1 mm, and 0.5 mm—were present in significantly lower proportions.

The proportion of non-granulated material (particles < 0.5 mm) ranged from 10.86% (for granulates containing 70% ECO-ZEC lime) to just 0.86% (for granulates with 15% PPR and 30% fly ash), indicating improved granulation efficiency with the latter formulation.

As noted by Marchuk et al. [76], particle diameter is a critical parameter influencing the uniform distribution of fertilizers by agricultural spreaders. According to the findings of Antille et al. [77], optimal granule diameters for spreader-based application range between 1.10 mm and 5.80 mm.

### 3.8. Disintegration Time of Fertilizer Granules in Water

The results of the disintegration time tests in water for fertilizer granulates, produced through the non-pressurized agglomeration of mixtures of carbonate limestone and the waste materials ECO-ZEC, PPR, and fly ash supplied by ROLPOL, are presented in Table 7.

Based on the conducted solubility tests (disintegration time in water), a notable influence of the waste materials ECO-ZEC, PPR, and fly ash on the disintegration time of the granulates was observed. Specifically, the addition of ECO-ZEC lime to ground carbonate lime resulted in a reduction in the disintegration time of the fertilizer granulates compared to the reference granulate made from carbonate lime alone.

**Figure 14:**
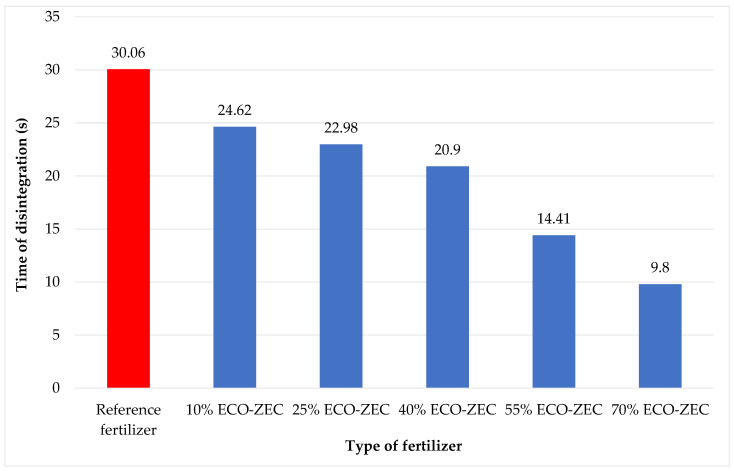
Disintegration time in water of granules obtained from mixtures of ECO-ZEC waste lime with ground lime.

Figure 14 shows the effect of ECO-ZEC waste lime on the solubility of fertilizer granulates.

The addition of 10% ECO-ZEC waste lime resulted in an approximate 18% reduction in the granule disintegration time (from 30.06 s for the reference granulate to 24.62 s for the granulate with 10% ECO-ZEC lime). Increasing the ECO-ZEC lime content from 10% to 70% further decreased the disintegration time by approximately 60%, reducing it from 24.62 s to 9.8 s.

A study by Vistoso et al. [78] examined the impact of fertilizer solubility on yield and pasture quality. Their findings indicated that fertilizers with higher solubility enhance nutrient availability to plants, leading to improved yields and feed quality. Similarly, Błaszczyk and Zakrzewska [79] emphasize that calcium fertilizers should exhibit high water solubility to facilitate the rapid neutralization of acidic pH in soils.

### 3.9. Statistical Analysis of Obtained Results

The hierarchical cluster analysis successfully categorized the tested pellets into five distinct clusters, labeled A–E, based on the material and process parameters of non-pressure agglomeration of carbonate limestone with the addition of biomass waste and selected binders.

Cluster A exhibits the highest contents of Na, Na_2_O, and pH. This cluster also includes pellets with the highest bulk density and kinetic durability, as well as the smallest percentage shares of the 0.5 mm and <0.5 mm fractions.

Cluster B, on the other hand, contains pellets with the highest contents of Ca and CaO. Pellets in this cluster also show the lowest bulk density and the smallest percentage shares of the 4.0 mm and 2.0 mm fractions, alongside the highest percentage share of the 8.0 mm fraction.

Cluster C is characterized by pellets with the lowest contents of Ca, CaO, S, and SO_3_ while exhibiting the highest contents of P, K, and heavy metals such as Cr, Mn, Fe, Co, Zn, Cd, and Pb. Additionally, pellets in this cluster have the lowest kinetic durability, the smallest percentage share of the 8.0 mm fraction, and the highest shares of the 4.0 mm, 2.0 mm, 0.5 mm, and <0.5 mm fractions.

Cluster D is distinguished by the highest contents of Mg, MgO, and N.

**Figure 15:**
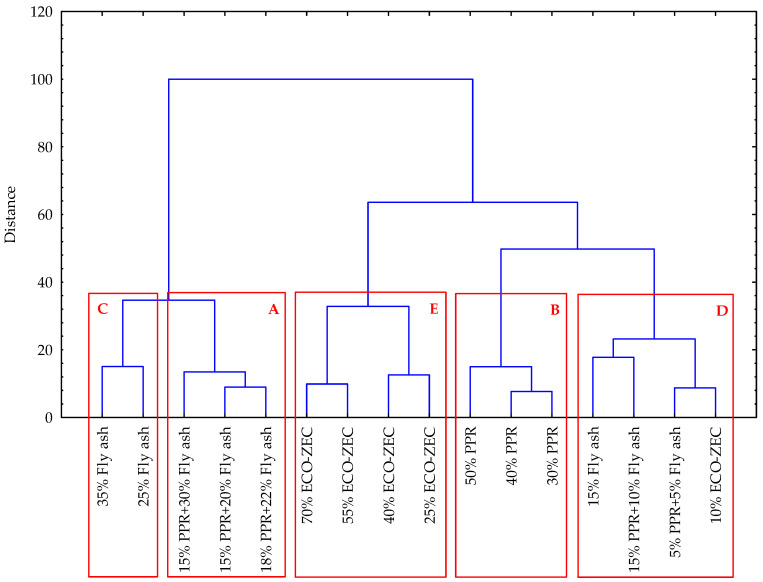
Dendrogram of hierarchical cluster analysis of pellets from carbonate limestone with the addition of biomass waste and selected binder.

Finally, Cluster E contains pellets with the lowest levels of Na, Na_2_O, Mg, MgO, N, P, K, and heavy metals (including Cr, Mn, Fe, Co, Zn, As, Cd, and Pb). This cluster is also marked by pellets with the highest contents of S and SO_3_, and the lowest pH (Figure 15).

Furthermore, when objects and features were grouped simultaneously, the analysis of material and process parameters for the non-pressure agglomeration of carbonate limestone with the addition of biomass waste and a selected binder revealed five distinctly dissimilar groups of objects.

The first group contained pellets characterized by the highest contents of Na, Na_2_O, pH, bulk density, and kinetic durability (red), along with the lowest percentage shares of the 0.5 mm and <0.5 mm fractions (green).

The second group consisted of pellets with the highest contents of Ca, CaO, and the largest percentage share of the 8.0 mm fraction (red). These pellets also exhibited the lowest bulk density and the smallest percentage shares of the 4.0 mm and 2.0 mm fractions (green).

The third group was characterized by pellets with the lowest contents of Ca, CaO, S, SO_3_, kinetic durability, and the percentage share of the 8.0 mm fraction (green). These pellets also had the highest contents of P, K, and heavy metals (e.g., Cr, Mn, Fe, Co, Zn, Cd, and Pb) (red). Moreover, pellets in this group, which had the highest percentage shares of the 4.0 mm, 2.0 mm, 0.5 mm, and <0.5 mm fractions (red), were grouped together.

The fourth group included pellets with the highest contents of Mg, MgO, and N (red).

**Figure 16:**
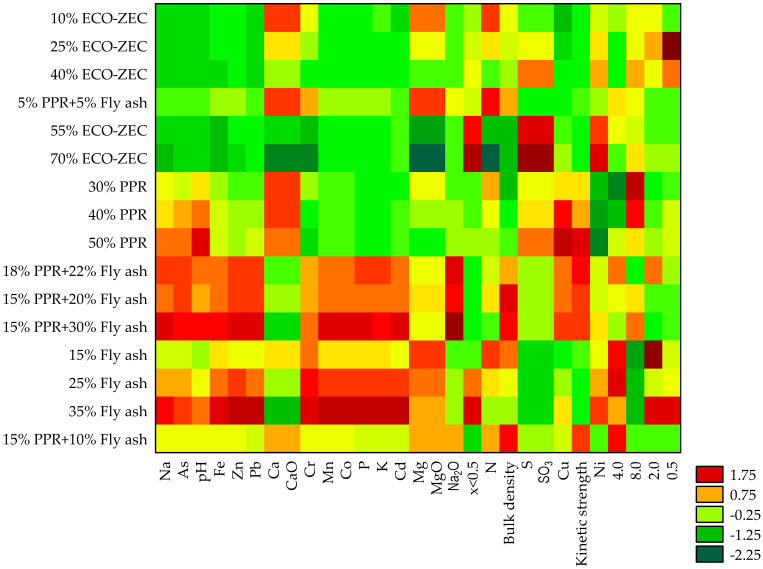
Graphical representation of results of simultaneous grouping of objects (pellets from carbonate limestone with the addition of biomass waste and selected binder) and features (material and process parameters of non-pressure agglomeration).

Finally, the fifth group consisted of pellets with the highest contents of S and SO_3_ (red), along with the lowest contents of Na, Na_2_O, Mg, MgO, N, P, K, heavy metals (e.g., Cr, Mn, Fe, Co, Zn, As, Cd, and Pb), and pH (green) (Figure 16).

PCA analysis facilitated the categorization of the analyzed pellets while preserving a significant portion of the explained variance. This approach condensed the variable set into two principal components (PC1 and PC2), indicating that the initial dataset of material and process parameters from the non-pressure agglomeration of carbonate limestone, combined with biomass waste and a selected binder, is highly correlated and therefore amenable to reduction.

**Figure 17:**
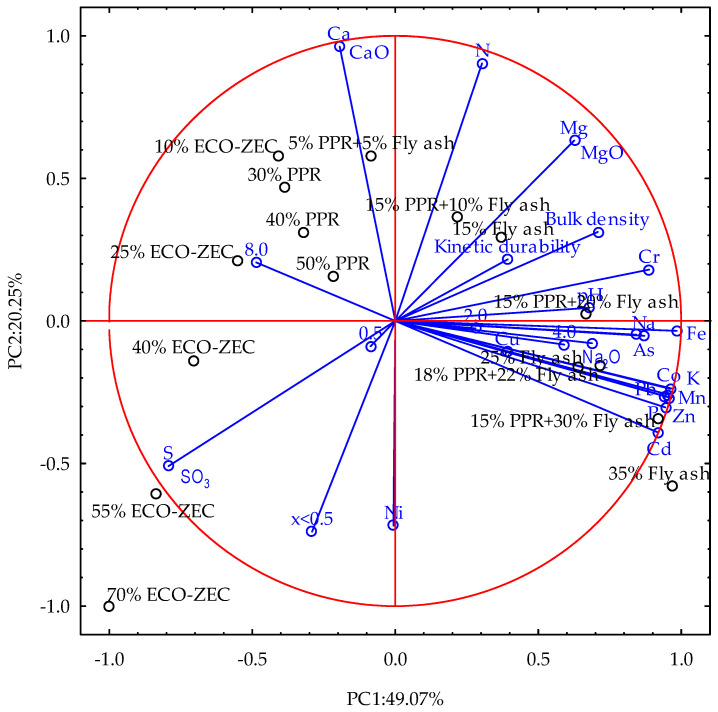
Biplot of material and process parameters of non-pressure agglomeration of carbonate limestone with the addition of biomass waste and selected binder showing first two principal components (PC1 and PC2) of PCA model that together explain 69.32% of total variance, i.e., 49.07% and 20.25% for PC1 and PC2, respectively. Blue biplot vectors indicate strength and direction of factor loading for all analyzed variables.

The first component (PC1) showed high negative loadings for the variables S and SO_3_ (−0.7928) and high positive loadings for Na, Na_2_O, and heavy metals, with values ranging from 0.6881 to 0.9844. In contrast, the second component (PC2) exhibited high negative loadings for Ni content and the percentage share of the <0.50 mm fraction (−0.7180 and −0.7390, respectively), while Ca, CaO, and N displayed high positive loadings, ranging from 0.9035 to 0.9623 (Figure 17).

## 4. Conclusions

The research conducted on the application of waste materials ECO-ZEC lime, PPR, and fly ash as additives in the production of lime-based fertilizer granules has provided valuable insights into their properties and potential for soil applications. The materials studied exhibited various physical and chemical characteristics, such as moisture content, particle size distribution, and elemental composition, which significantly influence the performance of the produced granules. The findings highlight the role of these additives in improving the functional properties of fertilizers, including their bulk density, crushing strength, dissolution time, and nutrient release patterns. Based on these observations, the following conclusions can be drawn:

1. The raw materials, including carbonate limestone and the waste additives ECO-ZEC, PPR, and fly ash supplied by ROLPOL, displayed relatively low moisture content. Specifically, unground lime had a moisture content of 0.36%, while fly ash and PPR additives exhibited slightly higher moisture values (0.40% and 1.82%, respectively), with ECO-ZEC lime showing the highest moisture content of 8.17%.
2. In terms of particle size distribution, ground lime had a significant proportion of fine particles, with 58.31% in the 0.25 mm fraction and 27.03% in the 0.125 mm fraction. Unground lime, on the other hand, contained larger particles, with 42.05% in the 1.00 mm fraction and approximately 19.39% in the 0.50 mm fraction.
3. The waste additives ECO-ZEC, PPR, and fly ash exhibited very fine particles, resulting in partial agglomeration during the sieving process, which could affect their incorporation into the final granules. These fine particles contributed to the unique characteristics of the granules produced.
4. When comparing bulk density values, unground lime exhibited a significantly higher bulk density (660.53 kg·m^−^^3^) than ground lime (460.78 kg·m^−^^3^). Among the waste additives, fly ash had the highest bulk density (536.35 kg·m^−^^3^), followed by ECO-ZEC lime (382.78 kg·m^−^^3^), and PPR, which had the lowest bulk density (262.44 kg·m^−^^3^).
5. Regarding chemical composition, fly ash exhibited the highest sodium content, while ECO-ZEC lime had the lowest. Magnesium content was highest in carbonate limestone, with fly ash containing approximately half as much and ECO-ZEC having the least. The calcium content in PPR was around 7% lower than in carbonate limestone, while ECO-ZEC and fly ash had 20% and over 30% less calcium, respectively.
6. The granules produced from the mixtures of carbonate limestone and waste materials ECO-ZEC, PPR, and fly ash showed CaO content ranging from 34% to 52%, indicating their potential for use as lime-based fertilizers. However, due to their low magnesium oxide (MgO) content, these granules cannot be classified as magnesium-containing lime fertilizers.
7. The granules exhibited low sodium content, with the highest levels found in the mixtures containing fly ash and the lowest in the ECO-ZEC mixtures. Additionally, the heavy metal content in the granules was within permissible limits, with fly ash mixtures exhibiting the highest concentrations of cadmium and lead.
8. The waste materials tested, ECO-ZEC, PPR, and fly ash, were highly alkaline, with pH values ranging from 12.37 for fly ash to 8.82 for the provided carbonate limestone. This alkaline nature indicates that the granules, when applied to soil, will not cause a reduction in soil pH, thus preventing potential acidification.
9. The addition of waste materials such as fly ash and PPR increased the potassium oxide (K_2_O) content, while the phosphorus pentoxide (P_2_O_5_) content remained moderate, and nitrogen content was relatively low. These changes in nutrient content could have an impact on the fertilizer’s suitability for various soil types and plant requirements.
10. The granules with 30% PPR exhibited comparable crushing strength (10.60 N) to the standard granule, while the addition of 50% PPR significantly increased the crushing strength to 14.00 N. The addition of ECO-ZEC lime to ground limestone caused a slight decrease in crushing strength, with values lower than those of the standard granule. However, the addition of fly ash resulted in a marked reduction in crushing strength, which could affect the granule’s durability.
11. Granules containing fly ash and PPR (above 10%) did not show disintegration during dissolution tests, indicating that these could be classified as slow-release fertilizers, potentially useful in applications where gradual nutrient release is required.

In conclusion, the results of this study provide a strong basis for the incorporation of waste materials, such as ECO-ZEC lime, PPR, and fly ash, into lime fertilizer formulations. The granules produced exhibited promising characteristics, including appropriate CaO content and beneficial nutrient profiles, making them suitable for use in soil applications. Furthermore, the incorporation of these waste materials contributes to the sustainability of fertilizer production, providing an avenue for recycling industrial by-products into valuable agricultural inputs.

## Figures and Tables

**Table 1:** Experimental design for the granulation process using mixtures of ground carbonate limestone and waste materials supplied by ROLPOL.

	Fertilizer Composition (%)			
	Limestone	ECO-ZEC	PPR	Fly Ash
10% ECO-ZEC	90	10	n/a	n/a
25% ECO-ZEC	75	25	n/a	n/a
40% ECO-ZEC	60	40	n/a	n/a
55% ECO-ZEC	45	55	n/a	n/a
70% ECO-ZEC	30	70	n/a	n/a
30% PPR	70	n/a	30	n/a
40% PPR	60	n/a	40	n/a
50% PPR	50	n/a	50	n/a
5% PPR + 5% Fly ash	90	n/a	5	5
18% PPR + 22% Fly ash	60	n/a	18	22
15% PPR + 10% Fly ash	75	n/a	15	10
15% PPR + 20% Fly ash	65	n/a	15	20
15% PPR + 30% Fly ash	55	n/a	15	30
15% Fly ash	85	n/a	n/a	15
25% Fly ash	75	n/a	n/a	25
35% Fly ash	65	n/a	n/a	35

n/a—not applicable (component not used in mixture).

**Table 2:** Moisture content of raw materials and waste additives ECO-ZEC, PPR, and fly ash.

Material	Moisture ± SD (%)
Ground lime	0.39 ± 0.02
Unground lime	0.36 ± 0.01
Fly ash	0.40 ± 0.01
PPR	1.82 ± 0.02
ECO-ZEC	8.17 ± 0.11

**Table 3:** Granulometric distribution of raw materials: limestone and waste materials ECO-ZEC, PPR, and fly ash subjected to sieve analysis.

Share of Fraction (%)						
Material	2.00	1.00	0.50	0.25	0.125	0.063
Ground lime	0.63	1.30	5.29	58.31	27.03	7.44
Unground lime	11.03	42.05	19.39	8.32	12.42	6.79
PPR	2.12	2.74	63.06	29.14	2.94	0.00
ECO-ZEC	20.70	26.50	52.79	0.00	0.00	0.00
Fly ash	0.00	0.06	0.52	48.14	31.32	19.96

**Table 4:** Results of analysis of macroelement content in raw material limestone and waste materials ECO-ZEC, PPR, and fly ash.

Material	Content of Macroelements, Sulfur, and Their Oxides (%)							
	Na	Mg	Ca	S	Na_2_O	MgO	CaO	SO_3_
Limestone	0.0130	1.1624	38.4384	0.0002	0.0172	1.9273	53.7753	0.0005
PPR	0.0926	0.1653	31.8823	15.2571	0.1228	0.2740	44.6033	38.1428
ECO-ZEC	0.0095	0.0360	18.2031	20.8159	0.0126	0.0597	25.4661	52.0398
Fly ash	0.1580	0.6787	3.2328	0.0251	0.2096	1.1253	4.5226	0.0628

**Table 5:** Results of analysis of macroelement content in the produced granulates.

Fertilizer Type		Content of Macroelements, Sulfur, and Their Oxides (%)							Fertilizer Variety
	Na	Mg	Ca	S	Na_2_O	MgO	CaO	SO_3_	
10% ECO-ZEC	0.0127	1.0498	36.4149	2.0818	0.0168	1.7405	50.9444	5.2044	05
25% ECO-ZEC	0.0121	0.8808	33.3796	5.2041	0.0161	1.4604	46.6980	13.0103	05
40% ECO-ZEC	0.0116	0.7118	30.3443	8.3265	0.0154	1.1802	42.4516	20.8162	05
55% ECO-ZEC	0.0111	0.5429	27.3090	11.4488	0.0147	0.9001	38.2053	28.6221	06
70% ECO-ZEC	0.0106	0.3739	24.2737	14.5712	0.0140	0.6200	33.9589	36.4280	07, D.1.
20% PPR	0.0289	0.9630	37.1272	3.0516	0.0384	1.5966	51.9409	7.6290	05
30% PPR	0.0369	0.8633	36.4716	4.5773	0.0489	1.4313	51.0237	11.4432	05
40% PPR	0.0449	0.7635	35.8160	6.1030	0.0595	1.2660	50.1065	15.2574	05
50% PPR	0.0528	0.6638	35.1603	7.6287	0.0700	1.1006	49.1893	19.0716	05
5% PPR + 5% Fly ash	0.0242	1.0884	36.3503	0.7643	0.1447	1.8045	50.8541	1.9107	05
18% PPR + 22% Fly ash	0.0592	0.8765	29.5131	2.7519	0.4380	1.4532	41.2888	6.8798	05
15% PPR + 10% Fly ash	0.0395	0.9645	33.9344	2.2912	0.2294	1.5991	47.4743	5.7281	05
15% PPR + 20% Fly ash	0.0540	0.9161	30.4139	2.2937	0.4039	1.5189	42.5490	5.7343	05
15% PPR + 30% Fly ash	0.0685	0.8677	26.8933	2.2962	0.5785	1.4387	37.6237	5.7405	06
15% Fly ash	0.0348	1.0898	33.1576	0.0039	0.0461	1.8070	46.3874	0.0098	05
25% Fly ash	0.0493	1.0415	29.6370	0.0064	0.0653	1.7268	41.4622	0.0161	05
35% Fly ash	0.0638	0.9931	26.1164	0.0089	0.0846	1.6466	36.5369	0.0223	06

**Table 6:** Results of analysis of microelement and heavy metal content in granulates obtained from mixtures of limestone and waste materials ECO-ZEC, PPR, and fly ash.

Fertilizer Type	Microelement and Heavy Metal Content (%)									
	Cr	Mn	Fe	Co	Ni	Cu	Zn	As	Cd	Pb
10% ECO-ZEC	1.14 × 10^−3^	9.27 × 10^−3^	1.20 × 10^−1^	5.63 × 10^−6^	5.56 × 10^−4^	1.01 × 10^−3^	2.51 × 10^−3^	2.46 × 10^−6^	1.81 × 10^−5^	1.56 × 10^−4^
25% ECO-ZEC	9.95 × 10^−4^	8.16 × 10^−3^	1.07 × 10^−1^	5.13 × 10^−6^	5.99 × 10^−4^	1.10 × 10^−3^	2.47 × 10^−3^	2.30 × 10^−6^	2.03 × 10^−5^	1.79 × 10^−4^
40% ECO-ZEC	8.32 × 10^−4^	6.88 × 10^−3^	9.14 × 10^−2^	4.54 × 10^−6^	6.48 × 10^−4^	1.20 × 10^−3^	2.43 × 10^−3^	2.12 × 10^−6^	2.29 × 10^−5^	2.05 × 10^−4^
55% ECO-ZEC	6.70 × 10^−4^	5.59 × 10^−3^	7.62 × 10^−2^	3.96 × 10^−6^	6.98 × 10^−4^	1.30 × 10^−3^	2.38 × 10^−3^	1.94 × 10^−6^	2.55 × 10^−5^	2.31 × 10^−4^
70% ECO-ZEC	5.07 × 10^−4^	4.31 × 10^−3^	6.09 × 10^−2^	3.37 × 10^−6^	7.47 × 10^−4^	1.40 × 10^−3^	2.34 × 10^−3^	1.76 × 10^−6^	2.80 × 10^−5^	2.57 × 10^−4^
20% PPR	1.05 × 10^−3^	9.91 × 10^−3^	1.61 × 10^−1^	9.98 × 10^−6^	4.11 × 10^−4^	1.69 × 10^−3^	3.99 × 10^−3^	4.39 × 10^−5^	2.29 × 10^−5^	4.28 × 10^−4^
30% PPR	9.57 × 10^−4^	9.91 × 10^−3^	1.61 × 10^−1^	9.98 × 10^−6^	4.11 × 10^−4^	1.69 × 10^−3^	3.99 × 10^−3^	4.39 × 10^−5^	2.29 × 10^−5^	4.28 × 10^−4^
40% PPR	8.24 × 10^−4^	9.74 × 10^−3^	1.73 × 10^−1^	1.16 × 10^−5^	3.65 × 10^−4^	2.01 × 10^−3^	4.61 × 10^−3^	6.16 × 10^−5^	2.59 × 10^−5^	5.54 × 10^−4^
50% PPR	7.13 × 10^−4^	9.60 × 10^−3^	1.84 × 10^−1^	1.30 × 10^−5^	3.27 × 10^−4^	2.28 × 10^−3^	5.13 × 10^−3^	7.63 × 10^−5^	2.83 × 10^−5^	6.59 × 10^−4^
5% PPR + 5% Fly ash	1.26 × 10^−3^	1.81 × 10^−2^	1.66 × 10^−1^	2.01 × 10^−5^	5.22 × 10^−4^	1.17 × 10^−3^	4.45 × 10^−3^	2.18 × 10^−5^	2.73 × 10^−5^	3.78 × 10^−4^
18% PPR + 22% Fly ash	1.28 × 10^−3^	4.46 × 10^−2^	2.76 × 10^−1^	6.71 × 10^−5^	5.55 × 10^−4^	1.86 × 10^−3^	1.07 × 10^−2^	8.13 × 10^−5^	6.49 × 10^−5^	1.16 × 10^−3^
15% PPR + 10% Fly ash	1.13 × 10^−3^	2.53 × 10^−2^	1.98 × 10^−1^	3.45 × 10^−5^	4.83 × 10^−4^	1.49 × 10^−3^	6.48 × 10^−3^	4.83 × 10^−5^	3.91 × 10^−5^	6.66 × 10^−4^
15% PPR + 20% Fly ash	1.29 × 10^−3^	4.46 × 10^−2^	2.76 × 10^−1^	6.71 × 10^−5^	5.55 × 10^−4^	1.86 × 10^−3^	1.07 × 10^−2^	8.13 × 10^−5^	6.49 × 10^−5^	1.16 × 10^−3^
15% PPR + 30% Fly ash	1.38 × 10^−3^	5.72 × 10^−2^	3.19 × 10^−1^	8.79 × 10^−5^	6.05 × 10^−4^	1.94 × 10^−3^	1.32 × 10^−2^	9.58 × 10^−5^	8.04 × 10^−5^	1.43 × 10^−3^
15% Fly ash	1.41 × 10^−3^	3.38 × 10^−2^	2.18 × 10^−1^	4.60 × 10^−5^	5.90 × 10^−4^	1.24 × 10^−3^	7.48 × 10^−3^	3.82 × 10^−5^	4.63 × 10^−5^	7.06 × 10^−4^
25% Fly ash	1.50 × 10^−3^	4.95 × 10^−2^	2.75 × 10^−1^	7.26 × 10^−5^	6.38 × 10^−4^	1.44 × 10^−3^	1.08 × 10^−2^	6.19 × 10^−5^	6.66 × 10^−5^	1.09 × 10^−3^
35% Fly ash	1.60 × 10^−3^	6.52 × 10^−2^	3.32 × 10^−1^	9.92 × 10^−5^	6.87 × 10^−4^	1.64 × 10^−3^	1.40 × 10^−2^	8.56 × 10^−5^	8.68 × 10^−5^	1.47 × 10^−3^

**Table 7:** Results of non-pressure agglomeration tests of mixtures containing carbonate lime and waste materials ECO-ZEC, PPR, and fly ash supplied by ROLPOL.

Type and Amount of Supplement	Compressive Strength (N)	Bulk Density (kg·m^−^^3^)	Granule Disintegration Time (s)	Granulometric Distribution (%)					
				Sieve Size (mm)					
				8	4	2	0.5	x < 0.5 Quantity of Non-Granulated Fraction	Quantity of Granulated Fractions
Mixtures with ground lime									
Reference fertilizer	10.60	1210.78	30.06	x	x	x	x	x	x
10% ECO-ZEC	5.11	1108.84	24.62	58.97	30.44	7.44	0.52	2.63	97.37
25% ECO-ZEC	4.99	1102.92	22.98	55.62	22.66	9.66	9.20	2.86	97.14
40% ECO-ZEC	4.85	1090.65	20.90	65.94	20.80	5.96	3.65	3.65	96.35
55% ECO-ZEC	4.76	1053.12	14.41	52.37	37.57	1.39	0.14	8.53	91.47
70% ECO-ZEC	4.65	1047.07	9.80	59.99	24.08	4.02	1.05	10.86	89.14
30% PPR	9.25	1054.74	x	93.18	5.34	0.25	0.19	1.04	98.96
40% PPR	10.76	1068.00	x	83.45	11.75	1.83	1.27	1.70	98.30
50% PPR	14.00	1084.00	x	60.24	31.30	4.23	1.75	2.47	97.52
5% PPR + 5% Fly ash	5.77	1129.77	912.67	54.21	39.91	2.01	0.45	3.43	96.58
18% PPR + 22% Fly ash	13.21	1137.77	x	38.48	49.44	10.58	0.62	0.87	99.12
15% PPR + 10% Fly ash	12.94	1162.38	x	40.95	56.22	2.47	0.22	0.14	99.86
15% PPR + 20% Fly ash	12.82	1172.43	x	59.74	38.64	1.42	0.00	0.20	99.80
15% PPR + 30% Fly ash	12.78	1159.72	x	70.79	28.11	0.14	0.10	0.86	99.14
15% Fly ash	5.57	1148.12	x	18.74	55.67	22.24	1.55	1.80	98.20
25% Fly ash	4.79	1112.19	x	26.46	59.75	5.04	1.94	6.81	93.19
35% Fly ash	4.60	1089.65	x	23.58	45.80	15.96	5.59	9.07	90.93

x—over 24 h.

## Data Availability

The original contributions presented in this study are included in the article. Further inquiries can be directed to the corresponding author.

## References

[1] Directive—1999/31—EN—EUR-Lex. https://eur-lex.europa.eu/eli/dir/1999/31/oj/eng.

[2] Act of 14 December 2012 on Waste. Dz. U. 2013 Poz. 21. https://isap.sejm.gov.pl/isap.nsf/DocDetails.xsp?id=wdu20130000021.

[3] Stępień M., Białecka B. (2017). Inwentaryzacja Innowacyjnych Technologii Odzysku Odpadów Energetycznych. Syst. Wspomag. Inż. Prod..

[4] GUS Rocznik Statystyczny Przemysłu 2019. https://stat.gov.pl/obszary-tematyczne/roczniki-statystyczne/roczniki-statystyczne/rocznik-statystyczny-przemyslu-2019,5,13.html.

[5] Vassilev S.V., Vassileva C.G., Baxter D. (2014). Trace Element Concentrations and Associations in Some Biomass Ashes. Fuel.

[6] Al-Fakih A., Mohammed B.S., Liew M.S., Nikbakht E. (2019). Incorporation of Waste Materials in the Manufacture of Masonry Bricks: An Update Review. J. Build. Eng..

[7] Mukhtar A., Qazi A.U., Khan Q.S., Munir M.J., Kazmi S.M.S., Hameed A. (2022). Feasibility of Using Coal Ash for the Production of Sustainable Bricks. Sustainability.

[8] (2012). Fly Ash for Concrete—Part 1: Definition, Specifications and Conformity Criteria.

[9] Zdunek A., Borowik M., Biskupski A. (2018). Nawozy z Popiołów. Chemia Przemysłowa.

[10] Belviso C. (2018). State-of-the-Art Applications of Fly Ash from Coal and Biomass: A Focus on Zeolite Synthesis Processes and Issues. Prog. Energy Combust. Sci..

[11] Vassilev S.V., Baxter D., Andersen L.K., Vassileva C.G. (2013). An Overview of the Composition and Application of Biomass Ash. Part 1. Phase–Mineral and Chemical Composition and Classification. Fuel.

[12] Yao X., Xu K., Li Y. (2016). Physicochemical Properties and Possible Applications of Waste Corncob Fly Ash from Biomass Gasification Industries of China. BioResources.

[13] Wójcik M., Stachowicz F., Masłoń A. (2020). The Use of Wood Biomass Ash in Sewage Sludge Treatment in Terms of Its Agricultural Utilization. Waste Biomass Valoriz..

[14] Zhai J., Burke I.T., Mayes W.M., Stewart D.I. (2021). New Insights into Biomass Combustion Ash Categorisation: A Phylogenetic Analysis. Fuel.

[15] Mercl F., Tejnecký V., Száková J., Tlustoš P. (2016). Nutrient Dynamics in Soil Solution and Wheat Response after Biomass Ash Amendments. Agron. J..

[16] Ochecová P., Mercl F., Košnář Z., Tlustoš P. (2017). Fertilization Efficiency of Wood Ash Pellets Amended by Gypsum and Superphosphate in the Ryegrass Growth Original Paper. Plant Soil Environ..

[17] Huygens D., Saveyn H., Tonini D., Eder P., Delgado Sancho L. (2019). Technical Proposals for Selected New Fertilising Materials under the Fertilising Products Regulation (Regulation (EU) 2019/1009). FeHPO CaHPO.

[18] Zhai J., Burke I.T., Stewart D.I. (2022). Potential Reuse Options for Biomass Combustion Ash as Affected by the Persistent Organic Pollutants (POPs) Content. J. Hazard. Mater. Adv..

[19] Rutkowska G., Wiśniewski K., Chalecki M., Górecka M., Miłosek K. (2016). Influence of Fly-Ashes on Properties of Ordinary Concretes. Ann. Wars. Univ. Life Sci. Land Reclam..

[20] Skels P., Haritonovs V., Pavlovskis E. (2021). Wood Fly Ash Stabilized Road Base Layers with High Recycled Asphalt Pavement Content. Balt. J. Road Bridge Eng..

[21] Kuźnia M., Zygmunt-Kowalska B., Szajding A., Magiera A., Stanik R., Gude M. (2022). Comparative Study on Selected Properties of Modified Polyurethane Foam with Fly Ash. Int. J. Mol. Sci..

[22] Kalembasa S., Godlewska A., Wysokinski A. (2008). Sklad Chemiczny Popiolow z Wegla Brunatnego i Kamiennego w Aspekcie Ich Rolniczego Zagospodarowania. Rocz. Glebozn..

[23] Łabętowicz J., Rutkowska B., Sosulski T., Stępień W., Szara E., Szulc W., Szymańska M. (2020). Nawozy z Odpadów Jako Źródło Składników Pokarmowych w Nawożeniu Roślin Uprawnych. Rolnicze Wykorzystanie Odpadów i Produktów Ubocznych Jako Ogniwo Gospodarki Obiegu Zamkniętego.

[24] Cruz N.C., Silva F.C., Tarelho L.A., Rodrigues S.M. (2019). Critical Review of Key Variables Affecting Potential Recycling Applications of Ash Produced at Large-Scale Biomass Combustion Plants. Resour. Conserv. Recycl..

[25] Trivedi N.S., Mandavgane S.A., Mehetre S., Kulkarni B.D. (2016). Characterization and Valorization of Biomass Ashes. Environ. Sci. Pollut. Res..

[26] Kohli S.J., Goyal D. (2010). Effect of Fly Ash Application on Some Soil Physical Properties and Microbial Activities. Acta Agrophys..

[27] Huotari N., Tillman-Sutela E., Moilanen M., Laiho R. (2015). Recycling of Ash–For the Good of the Environment?. For. Ecol. Manag..

[28] Shi R., Li J., Jiang J., Mehmood K., Liu Y., Xu R., Qian W. (2017). Characteristics of Biomass Ashes from Different Materials and Their Ameliorative Effects on Acid Soils. J. Environ. Sci..

[29] Paul S.C. (2020). Use of Fly Ash in Agriculture. Sustainable Agriculture.

[30] Manisha Basu M.B., Mahapatra S.C., Bhadoria P.B.S. (2006). Exploiting Fly Ash as Soil Ameliorant to Improve Productivity of Sabai Grass (*Eulaliopsis binata* (Retz.) CE Hubb) under Acid Lateritic Soil of India. Asian J. Plant Sci..

[31] Usmani Z., Kumar V., Gupta P., Gupta G., Rani R., Chandra A. (2019). Enhanced Soil Fertility, Plant Growth Promotion and Microbial Enzymatic Activities of Vermicomposted Fly Ash. Sci. Rep..

[32] Szostek M., Szpunar-Krok E., Jańczak-Pieniążek M., Ilek A. (2022). Short-Term Effect of Fly Ash from Biomass Combustion on Spring Rape Plants Growth, Nutrient, and Trace Elements Accumulation, and Soil Properties. Int. J. Environ. Res. Public Health.

[33] Mieldažys R., Jotautienė E., Jasinskas A. (2019). The Opportunities of Sustainable Biomass Ashes and Poultry Manure Recycling for Granulated Fertilizers. Sustainability.

[34] Kurzemann F.R., Fernandez-Delgado Juarez M., Probst M., Gómez-Brandón M., Spiegel H., Resch R., Insam H., Pötsch E.M. (2024). Biomass Ash as a Substitute for Lime and Its Impact on Grassland Soil, Forage, and Soil Microbiota. Agronomy.

[35] Paleckienė R., Sviklas A.M., Šlinkšienė R., Štreimikis V. (2012). Processing of Rape Straw Ash into Compound Fertilizers Using Sugar Factory Waste. Pol. J. Environ. Stud..

[36] Cruz N., Avellan A., Ruivo L., Silva F.C., Römkens P., Tarelho L.A.C., Rodrigues S.M. (2023). Biomass Ash-Based Soil Improvers: Impact of Formulation and Stabilization Conditions on Materials’ Properties. J. Clean. Prod..

[37] Drapanauskaitė D., Bunevičienė K., Repšienė R., Karčauskienė D., Mažeika R., Baltrusaitis J. (2022). The Effect of Pelletized Lime Kiln Dust Combined with Biomass Combustion Ash on Soil Properties and Plant Yield in a Three-Year Field Study. Land.

[38] Buneviciene K., Drapanauskaite D., Mazeika R., Tilvikiene V., Baltrusaitis J. (2021). Granulated Biofuel Ash as a Sustainable Source of Plant Nutrients. Waste Manag. Res. J. Sustain. Circ. Econ..

[39] Pesonen J., Kuokkanen V., Kuokkanen T., Illikainen M. (2016). Co-Granulation of Bio-Ash with Sewage Sludge and Lime for Fertilizer Use. J. Environ. Chem. Eng..

[40] Siuda R., Kwiatek J., Szufa S., Obraniak A., Piersa P., Adrian Ł., Modrzewski R., Ławińska K., Siczek K., Olejnik T.P. (2021). Industrial Verification and Research Development of Lime–Gypsum Fertilizer Granulation Method. Minerals.

[41] Bondarenko A., Tverda O., Repin M., Tkachuk K., Kofanov O., Kofanova O. (2021). The Use of Waste from the Production of Gravel as Fertilizer for Cultivation of Technical Energy Crops. Technol. Audit Prod. Reserv..

[42] Paleckienė R., Sviklas A.M., Šlinkšienė R. (2007). The Role of Sugar Factory Lime on Compound Fertilizer Properties. Pol. J. Environ. Stud..

[43] Ławińska K., Szufa S., Modrzewski R., Obraniak A., Wężyk T., Rostocki A., Olejnik T.P. (2020). Obtaining Granules from Waste Tannery Shavings and Mineral Additives by Wet Pulp Granulation. Molecules.

[44] Obraniak A., Gluba T., Ławińska K., Derbiszewski B. (2018). Minimisation of Environmental Effects Related with Storing Fly Ash from Combustion of Hard Coal. Environ. Prot. Eng..

[45] Gluba T.W., Obraniak A.Z., Siuda R.D., Kwiatek J.J. (2018). Sposób Wytwarzania Granulowanego Nawozu Wapniowego i/Lub Wapniowo-Magnezowego Pojedynczego Lub Wieloskładnikowego. Polish Patent.

[46] Gluba T.W., Obraniak A.Z., Siuda R.D., Kwiatek J.J., Olejnik T.P., Jabłoński A., Marszałek-Gubiec A.K., Pietrasik T. (2020). Sposób Wytwarzania Granulowanego Nawozu Wapniowego i/Lub Wapniowo-Magnezowego Pojedynczego Lub Wieloskładnikowego. Polish Patent.

[47] Nastaj S. (2010). Sposób granulacji aglomeracyjnej materiałów pylistych, zwłaszcza nawozów. Polish Patent.

[48] Corbett D., Lynch B., Wall D.P., Tuohy P. (2023). The Response of Finely Textured and Organic Soils to Lime and Phosphorus Application: Results from an Incubation Experiment. Soil Use Manag..

[49] (2023). Solid Biofuels—Determination of Moisture Content—Part 3: Moisture in the Sample for General Analysis.

[50] (2009). Feedstuffs.

[51] (2016). Solid Biofuels—Grain Composition of Crushed Pellets.

[52] Odzijewicz J.I., Wołejko E., Wydro U., Wasil M., Jabłońska-Trypuć A. (2022). Utilization of Ashes from Biomass Combustion. Energies.

[53] Czech T., Krupa A., Sobczyk A., Marchewicz A. (2017). Properties of fly ash derived from burning of industrial and medical waste and their impact on the environment. Inż. Ekol..

[54] Rosik-Dulewska: Podstawy Gospodarki Odpadami—Google Scholar. https://scholar.google.com/scholar_lookup?title=Podstawy+Gospodarki+Odpadami&author=Rosik-Dulewska,+C.&publication_year=2015.

[55] Jarosz-Krzemińska E., Poluszyńska J. (2020). Repurposing Fly Ash Derived from Biomass Combustion in Fluidized Bed Boilers in Large Energy Power Plants as a Mineral Soil Amendment. Energies.

[56] Regulation of the Minister of Economy of 8 September 2010 on the Method of Packaging Mineral Fertilizers, Placing Information on Fertilizer Components on Such Packaging, the Method of Testing Mineral Fertilizers and Types of Agricultural Lime. https://dziennikustaw.gov.pl/DU/2010/s/183/1229.

[57] Regulation of the Minister of Agriculture and Rural Development of 18 June 2008 on the Implementation of Certain Provisions of the Act on Fertilizers and Fertilization. https://isap.sejm.gov.pl/isap.nsf/DocDetails.xsp?id=wdu20081190765.

[58] Smołka-Danielowska D., Jabłońska M. (2022). Chemical and Mineral Composition of Ashes from Wood Biomass Combustion in Domestic Wood-Fired Furnaces. Int. J. Environ. Sci. Technol..

[59] Jukić M., Ćurković L., Šabarić J., Kerolli-Mustafa M. (2017). Fractionation of Heavy Metals in Fly Ash from Wood Biomass Using the BCR Sequential Extraction Procedure. Bull. Environ. Contam. Toxicol..

[60] Stankowski S., Chajduk E., Osińska B., Gibczyńska M. (2021). Biomass Ash as a Potential Raw Material for the Production of Mineral Fertilisers. Agron. Res..

[61] Balcarik L., Simackova B., Shaghaghi S., Syrova L. (2023). Effect of Ash from Biomass Combustion on Tailings pH. Eng. Proc..

[62] Feng C., Cheng M., Gao X., Qiao Y., Xu M. (2021). Occurrence Forms and Leachability of Inorganic Species in Ash Residues from Self-Sustaining Smouldering Combustion of Sewage Sludge. Proc. Combust. Inst..

[63] Gondal A.H., Hussain I., Ijaz A.B., Zafar A., Ch B.I., Zafar H., Sohail M.D., Niazi H., Touseef M., Khan A.A. (2021). Influence of Soil pH and Microbes on Mineral Solubility and Plant Nutrition: A Review. Int. J. Agric. Biol. Sci..

[64] Barrow N.J., Hartemink A.E. (2023). The Effects of pH on Nutrient Availability Depend on Both Soils and Plants. Plant Soil.

[65] Naz M., Dai Z., Hussain S., Tariq M., Danish S., Khan I.U., Qi S., Du D. (2022). The Soil pH and Heavy Metals Revealed Their Impact on Soil Microbial Community. J. Environ. Manag..

[66] Pandey B., Chen L. (2021). Technologies to Recover Nitrogen from Livestock Manure-A Review. Sci. Total Environ..

[67] Cwalina P., Obidziński S., Sienkiewicz A., Kowczyk-Sadowy M., Piekut J., Bagińska E., Mazur J. (2025). Production and Quality Assessment of Fertilizer Pellets from Compost with Sewage Sludge Ash (SSA) Addition. Materials.

[68] Gondek K., Kopec M. (2012). Zawartość Wybranych Makro i Mikroelementów w Przekompostowanych Komunalnych Odpadach Biodegradowalnych. Acta Agrophys..

[69] Wierzbowska J., Sienkiewicz S., Żarczyński P., Krzebietke S. (2020). Environmental Application of Ash from Incinerated Biomass. Agronomy.

[70] Latosińska J. (2020). Risk Assessment of Soil Contamination with Heavy Metals from Sewage Sludge and Ash after Its Incineration. Desalin. Water Treat..

[71] Miastkowski K., Leszczuk T., Bakier S. (2013). Zastosowanie Zawiesin Wodnych Bentonitu i Gliny Do Granulacji Bezciśnieniowej Nawozów Rolniczych. Inż. Apar. Chem..

[72] Malinowski P., Olech M., Sas J., Wantuch W., Biskupski A., Urbańczyk L., Borowik M., Kotowicz J. (2010). Production of Compound Mineral Fertilizers as a Method of Utilization of Waste Products in Chemical Company Alwernia S.A. PJCT.

[73] Mudryk K., Wrobel M., Jewiarz M., Niemiec M. Possibility of Using Chalk in Production of Mineral-Organic Fertilizers. Proceedings of the 17th International Scientific Conference “Engineering for Rural Development”.

[74] Balla Z., Tamás A., Vántus A., Hagymássy Z. (2017). Determining the Main Physical Characteristics of Fertilisers. Columella.

[75] Mudryk K., Frączek J., Wróbel M., Jewiarz M., Dziedzic K., Mudryk K., Werle S. (2018). Agglomeration of Ash-Based Fertilizer Mixtures from Biomass Combustion and Digestate. Renewable Energy Sources: Engineering, Technology, Innovation.

[76] Marchuk S., Tait S., Sinha P., Harris P., Antille D.L., McCabe B.K. (2023). Biosolids-Derived Fertilisers: A Review of Challenges and Opportunities. Sci. Total Environ..

[77] Antille D.L., Gallar L., Miller P.C., Godwin R.J. (2015). An Investigation into the Fertilizerparticle Dynamics Off-the-Disc. Appl. Eng. Agric..

[78] Vistoso E., Iraira S., Sandaña P. (2020). Effects of Phosphorus Fertilizer Solubility on Pastures Yield and Quality in Andisols. J. Soil Sci. Plant Nutr..

[79] Błaszczyk M., Zakrzewska M. (2017). Rola Inżynierii Chemicznej i Procesowej w Agrotechnice. Proceedings of the VII Seminarium Studenckie Bezpieczeństwo w Inżynierii Procesowej.

